# Clinicopathological Differences and Prognostic Value of Hypoxia-Inducible Factor-2a Expression for Gastric Cancer: Evidence From Meta-Analysis: Erratum

**DOI:** 10.1097/01.md.0000482826.94596.4d

**Published:** 2016-05-06

In the article “Clinicopathological Differences and Prognostic Value of Hypoxia-Inducible Factor-2a Expression for Gastric Cancer: Evidence From Meta-Analysis”,^1^ which appeared in Volume 95, Issue 7 of *Medicine*, several typos were reported. The article has since been corrected online.
